# Obituary: Kuan-Teh Jeang

**DOI:** 10.1186/1742-4690-10-28

**Published:** 2013-03-21

**Authors:** Ben Berkhout, Monsef Benkirane, Andrew Lever, Mark Wainberg, Ariberto Fassati, Persephone Borrow, Masahiro Fujii, Srimathy Sriskantharajah, Matthew Cockerill

**Affiliations:** 1 Department of Medical Microbiology, Laboratory of Experimental Virology, Center for Infection and Immunity Amsterdam (CINIMA), Academic Medical Center, University of Amsterdam, Meibergdreef 15, Amsterdam, 1105 AZ, The Netherlands; 2 Laboratoire de Virologie Moléculaire, Institut de Génétique Humaine, CNRS-UPR 1142, 141 rue de la Cardonille, 34296,, Montpellier, cedex 5, France; 3 Department of Medicine, University of Cambridge, Addenbrooke's Hospital, Cambridge, CB2 0QQ, UK; 4 McGill University AIDS Centre, Lady Davis Institute, Jewish General Hospital, Montreal, Québec, Canada; 5 The Wohl Virion Centre and MRC Centre for Medical & Molecular Virology, Division of Infection and Immunity, University College London, Cruciform Building, 90 Gower Street, London, WC1E 6BT, UK; 6 Nuffield Department of Clinical Medicine, University of Oxford, Weatherall Institute of Molecular Medicine, John Radcliffe Hospital, Headington, Oxford, OX3 9DS, UK; 7 Division of Virology, Niigata University Graduate School of Medical and Dental Sciences, Niigata, Japan; 8 BioMed Central, 236 Gray’s Inn Road, London, WC1X 8HB, UK

## Abstract

**Dear colleagues:**

Our loyal friend Kuan-Teh Jeang, “Teh” to friends and colleagues, passed away unexpectedly at the age of 54 on the evening of January 27, 2013. Great shock and sorrow was apparent in the avalanche of email messages by the very many international colleagues with whom Teh interacted over the years. Many of us came to know Teh as an energetic and gifted scientist for whom we had much respect and affection.

## 

Teh (Figure 1) was born in 1958 in Taichung, Taiwan and was the youngest to his two older brothers. Teh spent his childhood in Libya and came to the US in 1970. At age 16, he began college at the Massachusetts Institute of Technology, and after two years, started medical school at Johns Hopkins University, receiving both his M.D. and Ph.D. degrees by age 25. His Ph.D. thesis was on the regulation of gene expression in cytomegalovirus with Dr. Gary S. Hayward as advisor. During his time at Hopkins, Teh met his wife, Diane, a graduate student in the same laboratory. They married in 1984 in Iowa, where Teh completed his medical internship. The next year, Teh started his post-doctoral work at the National Institutes of Health (NIH) in the laboratory of Dr. George Khoury at the National Cancer Institute. Dr. Khoury died much too early at the age of 43 in 1987, but he was Teh’s role model and influenced him greatly in his professional life. As a recognition of his scientific achievements, Teh was recently selected to deliver the 2012 George Khoury Lecture at NIH on cellular transformation by the human T cell leukemia virus (HTLV-I).

**Figure 1:**
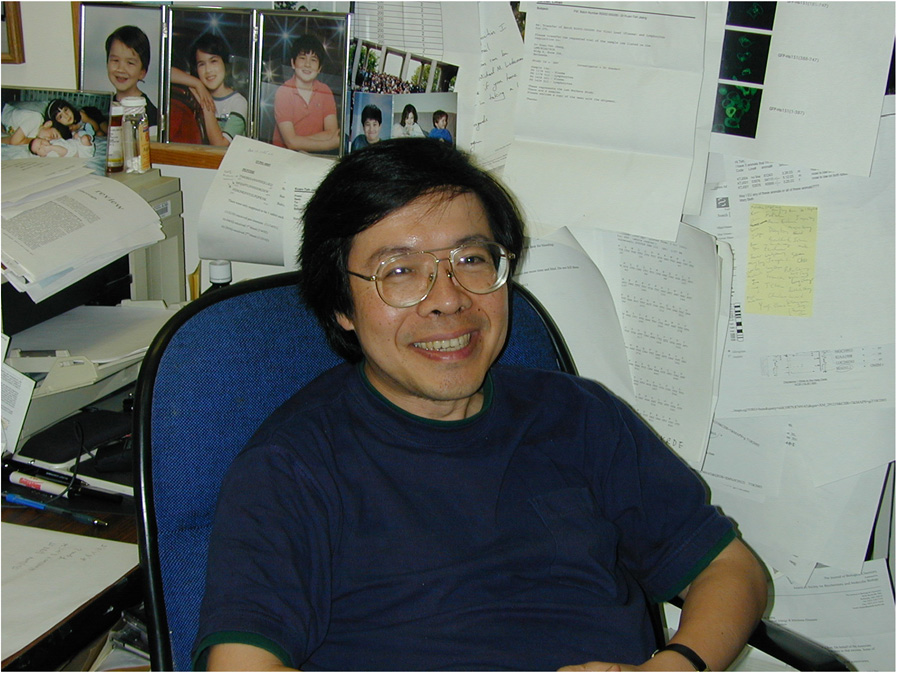
Dr Kuan-Teh Jeang.

Teh had been working at the NIH in Bethesda for 27 years, exactly half of his life, and was chief of the Molecular Virology Section in the Laboratory of Molecular Microbiology. His major research interest was around the human immunodeficiency virus (HIV-1) and HTLV-I, with an abundant production of more than 300 scientific publications on the molecular details of virus replication and relevant disease-causing mechanisms of viral pathogenesis. Teh was a true experimentalist with an interest in the implementation of new technologies to get to the next level of understanding of the biology of human pathogenic viruses. He only stopped bench work in 2004 when he became editor-in-chief of *Retrovirology*.

HTLV-1 is linked to the development of adult T-cell leukemia and a variety of inflammatory manifestations including HTLV-1 associated myelopathy. Teh was the first to show that HTLV-1 transcription is regulated through the cAMP signaling pathway implicating roles for CREB and CBP before these proteins were clearly identified and cloned. His research team also contributed to our understanding of how the viral Tax oncoprotein activates the pro-inflammatory factor NF-kB. More recently, he proposed a role for deubiquitinases in the regulation of TRAF6–mediated NF-kB signaling. Teh’s work has also advanced our understanding of genetic damage in virus-cellular transformation. In 1990, he first reported that the HTLV-1 Tax oncoprotein repressed DNA-repair. Thereafter, he characterized the important roles of dysregulated mitotic checkpoint and AKT activation in cellular transformation. His work has contributed to the elucidation of the role played by the spindle assembly checkpoint in oncogenesis, helping to explain how the loss of multiple checkpoints alters cancer tropism in vivo.

Teh had a long standing interest in understanding the viral and cellular factors that govern HIV-1 gene expression in infected human cells. In the late 1980s, Teh’s lab showed that HIV-1 uses an unprecedented mechanism of transcription that is dictated by an RNA-binding protein, Tat, which binds a nascent viral RNA target (TAR), the first RNA enhancer element ever described. Subsequently, Teh’s group characterized cellular RNA-binding proteins that regulate HIV-1 replication, including the TAR RNA binding protein (TRBP) that later became known as an important factor of the cellular RNA interference machinery. In recent work, his lab completed a genome-wide screening for human cell factors that are needed for HIV-1 replication. Using novel technology, Teh extended his interests in RNA-biology through the identification of small RNAs (i.e. siRNAs and miRNAs) that have biologically important roles in viral infection, cellular metabolism and virus-induced pathogenesis.

In addition to all these accomplishments, one of Teh’s greatest contributions to science probably lies in his role as mentor for young scientists. Teh trained 37 international postdoctoral fellows and 7 more are currently working in his group at the NIH. He has been a fantastic mentor of young scientists who have since spread across the globe, from Taiwan to China, from France to the Netherlands, from Canada to many places in the United States. Many flew into Washington DC to attend the funeral ceremony on February 9^th^. Teh was attentive, supportive and kind, but at the same time, demanding to his postdocs. Importantly, he set himself as an unselfish role model for them, working essentially 7 days per week and regular working days ran from 7 am to 7 pm. But, also during the evening and at night, one never had to wait long for reply emails. He was truly interested and at many times influenced the future careers of those who were lucky enough to have spent some time in his laboratory. In fact, his words of advice reached many more individuals with whom he came across in his professional and private life. His mentoring commitment is also reflected in his service to many professional societies. For instance, Teh was a standing member of the AIDS Molecular and Cellular Biology (AMCB) Study Section, where he had a reputation for being strongly supportive of new investigators.

Teh always had a special interest in the area of scientific publication. For instance, in 1994 he joined the editorial board of the *Journal of Biomedical Science* (JBS) of the National Science Council of Taiwan, the country in which he was born. He was an avid advocate for ways to improve the journal’s Impact Factor. He left this journal in 2004 to free himself for an important new activity: the launch of the journal *Retrovirology*, where he served as Editor-in-Chief since the journal’s inception. From the earliest years, Teh was an advocate of the Open Access publishing format. In particular, at the helm of *Retrovirology*, Teh witnessed much skepticism in regard to Open Access publishing during the early years and indomitably championed the new publishing format while overcoming initial prejudice. Teh often referred to himself and his Editorial Board members as the ‘Young Turks’ of virology, pioneering a new and exciting publishing format. Unsurprisingly, his talent for kicking off new initiatives paid off and *Retrovirology* is currently among the highest cited journals in the field of virology, and all of this was achieved in less than 10 years. In addition, he served on the editorial board of numerous journals, including the *Journal of Virology* and the *Journal of Biological Chemistry*.

Teh – as all who knew him will attest – would never rest on his laurels and although *Retrovirology* had become a leader in its field, he pushed for more journal involvement with the retroviral community .… and Frontiers in Retrovirology was born in 2009. It was the first conference organized by a BioMed Central journal – another example of Teh taking the lead – and proved to be such a universal success that the 3^rd^ conference in the series will take place in September 2013 in the UK in Cambridge.

Teh was a scientist with a vision and a broad interest in all aspects of scientific endeavor. He also was a true scientific leader, initiating scientific debate, writing editorials, sitting on many committees, orchestrating new book volumes and organizing international meetings on diverse topics. For instance, he was president of the Society of Chinese Bioscientists in America (SCBA) in 2010 and voiced the strong opinion that the representation of Asian-American scientists in leadership positions should be increased. He was instrumental in the decision to take the 2011 SCBA conference to China.

He was the recipient of an extraordinary number of awards, most recently the International Retrovirology Association’s Dale McFarlin Award in 2011, BioMed Central’s Open Access “Editor of the Year” in 2010 and the John’s Hopkins University Woodrow Wilson Award in 2009. Teh was also elected to membership of prestigious societies such as the Academia Sinica in Taiwan, Fellowship in the American Academy of Microbiology, and Fellowship in the American Association for the Advancement of Science.

Teh had an infectious enthusiasm and winner’s mentality both at work and play. He was a skilled tennis and chess player, a gifted writer, and a great debater with strong opinions on virtually all subjects of science and life in general. Additionally, he had a passion for current events and a love of travel, movies, food and music.

Teh’s death is a blow to the retrovirus research community and we will sorely miss his scientific leadership. He has been central to so much of what we have accomplished together as well as being a supportive and generous friend to many of us individually. Teh’s life was much too short, but his legacy and our memories of him will last forever. Our hearts and condolences are with his wife Diane and his three children David (23), Diana (20), and John (15).

## A note from the Publisher

Kuan-Teh Jeang was one of BioMed Central’s first academic Editors-in-Chief, launching *Retrovirology* on our platform in 2004. From the very beginning, his level of enthusiasm and energy was extraordinary, and the results he achieved in developing *Retrovirology* showed he was just as much of a dynamo as an Editor as he was in the laboratory.

Teh was a friend and close colleague to many at BioMed Central, past and present. His passing at such a young age caused shock and great sadness. He was simultaneously one of our most solid friends and supporters, while also being a tireless force driving change and improvement, reminding us not to sit on our laurels, and always ready with forthright but constructive criticism when he thought we needed to do better.

Teh took a “24x7” approach, and he encouraged those he worked with at BioMed Central to do the same. In return, he was generous with praise and each Christmas he would recognize the BioMed Central team which supported *Retrovirology* with thanks and gifts. Teh’s determination to make *Retrovirology* the leading journal in its field was striking, and his journal’s continuing progress up the impact factor rankings gave him huge satisfaction and pride, culminating in June 2012 when *Retrovirology* overtook *Journal of Virology*.

Teh recognized that, for open access to the results of scientific research to become a reality, a viable business model was crucial, and so he was always supportive of BioMed Central’s growth as a publisher. Introductions made by Teh helped BioMed Central to build its journal portfolio, especially in Asia, with *Journal of Biomedical Science* and *Cell and Bioscience.*

The success of *Retrovirology* demonstrated that a strong Editor-in-Chief could build a scientific community around an open access journal. Teh encouraged BioMed Central to take this community-building into the physical realm by arranging a scientific conference in association with the journal. The 3rd *Frontiers of Retrovirology* conference will be held this year, and the event has provided a template for BioMed Central’s growing number of successful journal-associated conferences.

**Figure 2:**
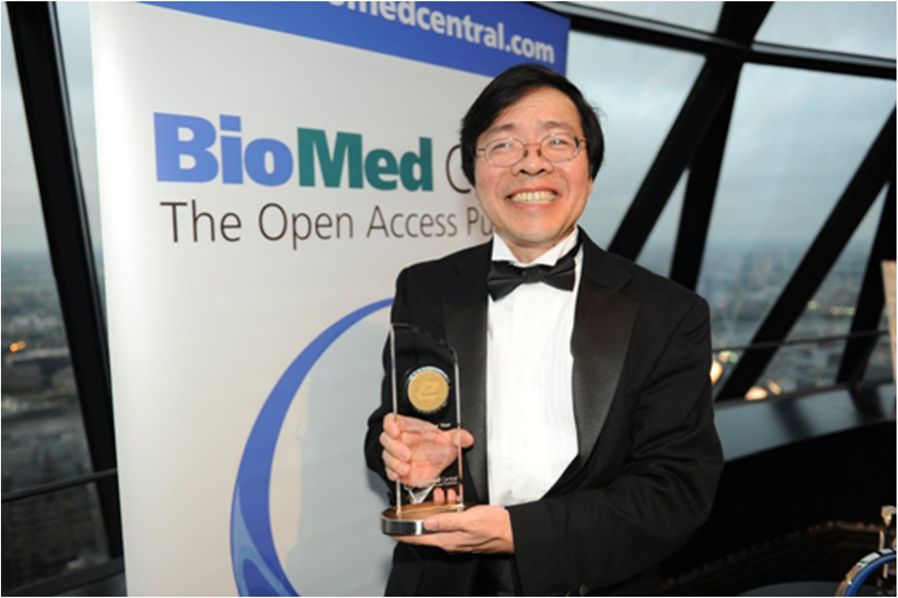
Teh with his Editor-of the Year Award.

Teh was a deserved winner of our 2010 Editor of the Year award, which he celebrated in London at BioMed Central’s 10^th^ anniversary party (Figure 2). He will be sadly missed by all of us.

Matthew Cockerill

Managing Director, BioMed Central

